# Ultrasensitive and Cost-Effective Detection of Neuropeptide-Y by a Disposable Immunosensor: A New Functionalization Route for Indium-Tin Oxide Surface

**DOI:** 10.3390/bios12110925

**Published:** 2022-10-26

**Authors:** İnci Uludağ, Mustafa Kemal Sezgintürk

**Affiliations:** Bioengineering Department, Faculty of Engineering, Çanakkale Onsekiz Mart University, Çanakkale 17100, Turkey

**Keywords:** Neuropeptide Y, indium tin oxide-coated polyethylene terephthalate, hexamethylene diisocyanate, electrochemical impedance spectroscopy, neurodegenerative disease, neuroimmune disorders

## Abstract

Neuropeptide Y (NPY) is one of the most abundant neuropeptides in the human brain, and its levels in the blood change in neurodegenerative and neuroimmune disorders. This indicates that NPY may serve as a diagnostic and monitoring marker for associated disorders. In this paper, an electrochemical immunosensor was created to detect NPY biomarkers using a novel immobilization technique. The proposed biosensor system enables accurate, specific, cost-effective, and practical biomarker analysis. Indium tin oxide-coated polyethylene terephthalate (ITO-PET) sheets were treated with hexamethylene diisocyanate (HMDC) to covalently immobilize antibodies. Electrochemical impedance spectroscopy (EIS) and cyclic voltammetry (CV) techniques were used to analyze each step of the biosensors. The proposed NPY biosensor has a broad linear detection range (0.01–100 pg mL^−1^), a low limit of detection (LOD) (0.02968 pg mL^−1^), and a low limit of quantification (LOQ) (0.0989 pg mL^−1^). Atomic force microscopy (AFM) was used to support in the optimization process, study the surface morphology, and visualize it. Studies of repeatability, reproducibility, storage, and Kramers–Kronig transformation were conducted during electrochemical characterization. After analytical experiments, the biosensor’s responses to human serum samples were evaluated. According to the obtained data, the error margin is small, and the created biosensor offers a great deal of promise for the clinical measurement of NPY.

## 1. Introduction

Biosensors are utilized for early illness detection, patient health monitoring, tracking disease progression, and managing human health. The primary components are a molecular recognition element and a transducer. The molecular recognition elements most often include whole cells, receptors, organelles, antibodies, tissues, microbes, enzymes, molecularly imprinted polymers, and peptide nucleic acids. In electrochemical biosensors, the concept of detection is based on the transducer element transforming the biological sensing element’s response to a specific analyte into an electrical signal [1]. 

EIS is an effective method for examining electrochemical systems and processes. EIS measures the frequency-dependent alternating current. This information is used to compute the charge transfer resistance (R_ct_). The R_ct_ value is altered by interactions between the biorecognition element and the analyte. Analyte interactions at the surface of the modified electrode alter the interfacial electron transfer resistance between the electrode and an electroactive species in a solution that is reversibly reduced and oxidized to produce an alternating current. The form of the EIS spectrum shows the electrochemical system’s diffusional properties and charge transfer kinetics. The circuit analogous to the Randles–Ershler model is the most prevalent design. This model consists of R_ct_, electrolyte resistance (Rs), double-layer capacitance (Cdl), and a diffusional resistance component (Warburg impedance). R_ct_ represents the kinetics of charge transfer. The Rs parameter is determined using the distance between the electrodes and the solution’s conductivity. The capacitance of a double layer is dependent on the electrode area and ion concentration. These constants have particular values in the Randles–Ershler equivalent circuit for the EIS spectrum that provides the greatest match [2,3]. Additionally, EIS may identify an electrode’s physical properties, such as surface roughness and porosity [4].

Neuropeptide Y (NPY) is a 36-amino acid peptide abundant in the brains of mammals. It functions as a neurotransmitter, modulator, and nervous system regulator [5]. It regulates food intake, metabolic function, circadian rhythm, neuronal excitability, addiction, and emotional reactions to diverse stimuli [6]. Concentrations of NPY vary dramatically across neurodegenerative and neuroimmune diseases. This situation shows that NPY may be beneficial in detecting and monitoring related diseases. Several studies demonstrate that several neuropeptides are critical in the etiology of Alzheimer’s, Parkinson’s, and autism illnesses [5,7,8]. Additionally, anxiety and depression significantly increase the secretion of NPY [6,9]. Cheng et al. found that chronic unexpectedly mild stress (CUMS) increased NPY expression in prostate cancer cells [9]. Variations in NPY levels have also been seen in illnesses characterized by dopaminergic neuron degeneration, such as Parkinson’s disease (P). In animal models of P, nigrostriatal dopamine deprivation has been shown to result in a considerable increase in the number of NPY-expressing cells. In addition, the proportion of interneurons expressing NPY mRNA was considerably greater in P patients than in controls [5]. Moreover, psychological depression has been reported to increase tumor-associated macrophages in the prostate tumor microenvironment, resulting in increased NPY expression. The increased tumor-associated macrophages further activate an IL6–STAT3 signaling pathway in prostate cancer cells and promote prostate cancer growth. Consequently, there is a strong correlation between an elevated NPY level and cancer development, making early disease detection crucial [9].

The present study created a label-free immunosensor based on ITO films coated on a flexible PET to detect NPY. We activated the ITO-PET surface with HMDC for NPY detection. HMDC is the most commonly used aliphatic diisocyanate [10,11]. The N=C=O groups of diisocyanates react effectively with almost no by-products with biological macromolecules containing nucleophilic –NH, –SH, –COOH, and OH polyol groups. The activation of the ITO-PET surface with hydroxyl groups is followed by the reactivity of the HMDC isocyanate groups with the surface during the manufacturing process of the proposed electrode. Isocyanate groups already present on the modified ITO-PET surface enable the immobilization of antibodies. Using CV and EIS in the presence of Fe(CN)_6_^4−/3−^ as a redox-active species, the interfacial characteristics of the modified ITO-PET electrode were evaluated, and different manufacturing process parameters were adjusted. In addition to these electrochemical techniques, atomic force microscopy (AFM) was employed to determine the morphology of the surfaces after immobilization. As a result, we have developed an immunosensor device that is effective and sensitive to NPY biomarker.

## 2. Material and Methods

### 2.1. Chemicals and Equipment

All electrochemical studies were conducted using a Gamry potentiostat/galvanostat (Reference 600, Gamry Instruments, Warminster, PA, USA), and electrochemical measurements were made using a 5 mM [Fe(CN)_6_] ^3−/4−^ 50 mM PBS solution containing 0.1 M KCl as a redox probe (pH 7.4). Sigma Aldrich supplied all chemicals and ITO-PET films (USA). For electrochemical studies, ITO-PET electrodes (2 mm × 20 mm) were utilized as working electrodes in a triple electrode system. An Ag/AgCl reference electrode and a platinum wire counter electrode were also employed. The Elga LC134 system (18.2 M cm^−1^) produced ultrapure water. Polyclonal Anti-NPY Antibody from rabbit (Product number: N9528) and NPY human protein (Product number: N5017) were purchased from Sigma Aldrich, St. Louis, MO, USA. Both were prepared in phosphate buffer at pH 7. Human serum samples were purchased from Sigma-Aldrich (Product numbers: H4522, H6914, H3667, S1-100ML) and Thermo fisher (Product number: 31876). Witec Alpha 300RA brand AFM was used at the Canakkale Onsekiz Mart University (COBILTUM) Scientific and Technological Research Center to analyze the surface morphological changes that occur throughout the immobilization phases of the biosensor.

### 2.2. Electrochemical Analyses

Electrodes that had experienced all immobilization processes were treated with standard NPY solutions of concentrations ranging. Electrochemical impedance spectroscopy and cyclic voltammetry measurements characterize the surface behavior at different NPY concentrations and each optimization stage. Electrochemical measurements were performed inside the redox probe. For CV measurements, the applied voltage was set between 0.5 V and 5 V, with a step size of 10 mV and a sweep rate of 100 mV s^−1^. The formal impedance measuring potential is 0 V and 5 mV for alternating current. The impedance was measured between 50,000 and 0.05 Hz. 

### 2.3. Development of a Biosensing System for the Analysis of Neuropeptide Y

**Scheme 1:**
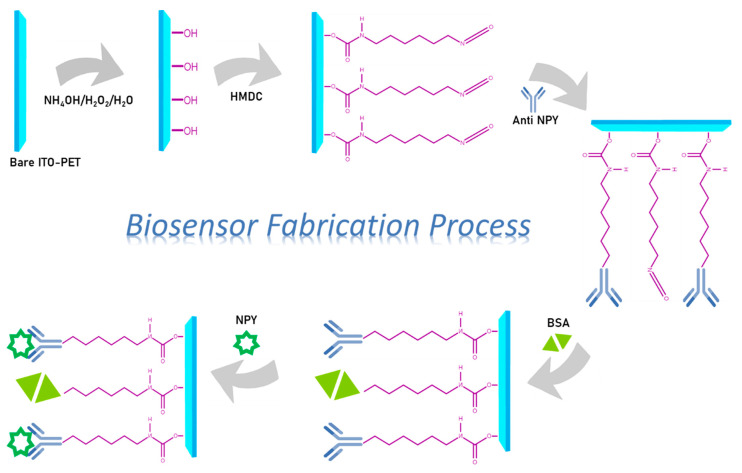
The processes involved in immobilizing the NPY immunosensor.

As illustrated in Scheme 1, the manufacturing of immunosensors consisted of four distinct steps: cleaning and activation of the ITO-PET surface, modification of the electrode surface with HMDC, antibody immobilization, and protein analysis. Ultrasonication was used to clean ITO-PET electrodes for 10 min in acetone, soap solution, and ultrapure water. After cleaning, the electrodes were put in a solution of ammonium hydroxide (NH_4_OH), hydrogen peroxide (H_2_O_2_), and ultrapure water (1:1:5) for 90 min to create hydroxyl groups on the surface. Simultaneously, a 0.5 percent HMDC solution (in acetone) was produced. It is required that the solution be freshly prepared. After hydroxylation, the electrodes were incubated in HMDC solution for 45 min. After removing physically adsorbed HMDC molecules from the ITO-PET electrodes by washing them with acetone and ultrapure water, they were dried in argon gas. The interaction of cyanate groups with hydroxyl groups (OH) on the surface of the electrode led to the formation of a suitable surface for immobilizing an antibody. Each electrode was treated with 200 µL of a 62.8 ng mL^−1^ anti-NPY solution for 45 min to immobilize the antibodies. Following incubation with NPY antibodies, the electrodes were washed with ultrapure water to remove unattached antibody molecules. In order to prevent non-specific binding to the electrode surface, the electrodes were then treated with 0.5% BSA for 60 min and then washed with ultrapure water. This biosensor was stored at +4 °C until NPY antigen testing was conducted. 

Adjustments were performed to the HMDC and anti-NPY concentrations and incubation times, and the NPY incubation period to enhance the biosensor’s sensitivity and repeatability. The biosensor’s ability to detect increasing NPY solution concentrations was evaluated throughout the optimization process, and impedance-based calibration graphs were developed.

Evaluating the linear detection range of the proposed NPY biosensor, the fabricated biosensors were incubated under conditions optimized for increasing NPY concentrations. Then, EIS and CV measurements were made. The R_ct_ varied with the concentration, and a calibration graph for the proposed biosensor was generated using the equivalent circuit model. Simultaneously, the Kramers–Kronig transformation was used to evaluate if the NPY biosensor’s impedance spectrum had been deviated by external influences. The standard deviation under parameters of repeatability is a characteristic of precision and accuracy. This was performed by incubating 20 distinct electrodes manufactured under identical conditions with the same concentration (25 pg mL^−1^) of NPY and measuring their EIS properties. The generated data were examined statistically. Standard deviation, mean value, and coefficient of variation calculations were undertaken. In addition, the responses of NPY biosensors created under similar conditions but at different times and by different persons were compared. This method was repeated 10 times to assess the reproducibility of the biosensor.

In addition, the susceptibility of the NPY biosensor to human serum samples was assessed by analyzing five different human serum samples. The serum concentration of NPY was determined using the standard addition technique. According to reports, the normal concentration of NPY in human plasma is between 0.25 and 129 pM. [12]. To analyze the serum samples within the linear range of the proposed biosensor, they were diluted by a factor of 1000. For standard addition, 5 pg mL^−1^ and 25 pg mL^−1^ NPY concentrations were used. The experiment-derived impedance values were computed using the equation from the calibration table.

## 3. Results 

**Figure 1:**
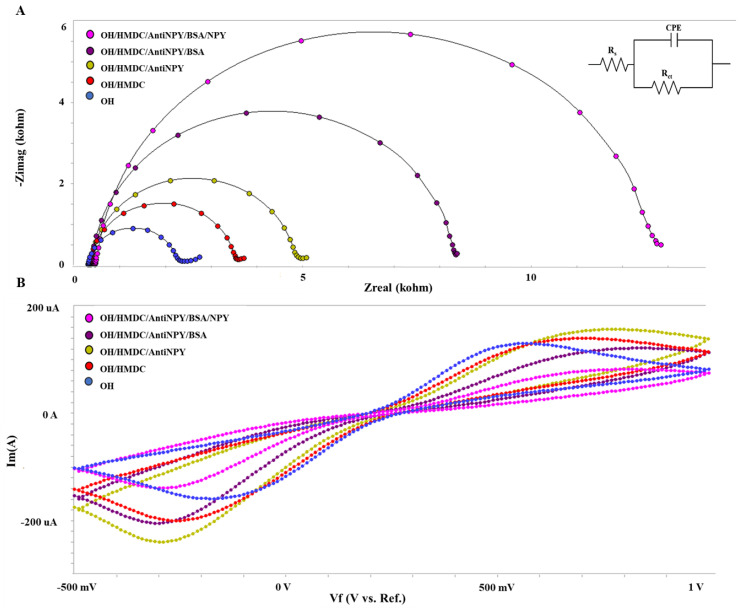
(**A**) EIS curves and (**B**) CV voltammograms of the biosensor’s immobilization phases for NPY determination.

EIS is an effective and highly sensitive method for monitoring the impedance change of the biosensor during modification processes. This essential tool provides data on the interfacial characteristics between the electrode surface and electrolyte solution, the adsorption process, and the interactions of biorecognition molecules with the electrode surface. Figure 1 demonstrates the impedance spectra of successive construction used in this work.

Due to the electrode surface modifications throughout the immobilization stages, the electrode surface varies in thickness and conductivity. Based on these changes, EIS measurements identify the target analyte instantaneously. The semicircular region dominates the EIS spectrum, indicating the R_ct_ at higher frequencies and the Warburg impedance at lower frequencies. During the first immobilization stage, self-assembled monolayers were formed on the electrode surface by covalently attaching OH groups to the ITO-PET surface.

**Figure 2:**
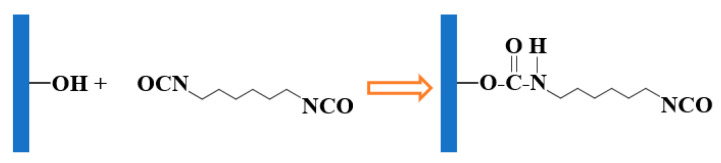
HMDC treatment of the hydroxylated electrode surface.

After the electrode surface was changed with HMDC, the R_ct_ value increased compared to the previous Nyquist curve. HMDC modification prepares the surface for the subsequent immobilization of antibodies. Figure 2 depicts the covalent connection between the OH groups on the hydroxylated electrode surface and HMDC. Due to the creation of an extra layer on the electrode surface, it became difficult for the redox probe to diffuse to the electrode surface. At this time, both the R_ct_ value and the diameter of the Nyquist semicircle increased (Figure 1A). The surface of the electrode was then coated with anti-NPY. The cyanates groups, which may react rapidly with functional groups found in proteins, generated covalent bonds with anti-NPY. BSA (0.5 percent) was utilized as a blocking agent to remove non-interacting active ends. On the insulating electrode’s surface, the charge transfer resistance continued to expand. 

Finally, when rising antibody–antigen concentrations were found within the detection range, moderate antibody–antigen interactions occurred on the electrode surfaces. The R_ct_ value was shown to increase with increasing antibody–antigen concentrations.

In addition to EIS data through all immobilization stages, CV measurements of electrode surface changes were also obtained. Figure 1B displays CV voltammograms for all immobilization phases of the NPY-measuring biosensor. When the insulation on the surface of the electrode is increased, peak currents are observed to decrease considerably.

**Figure 3:**
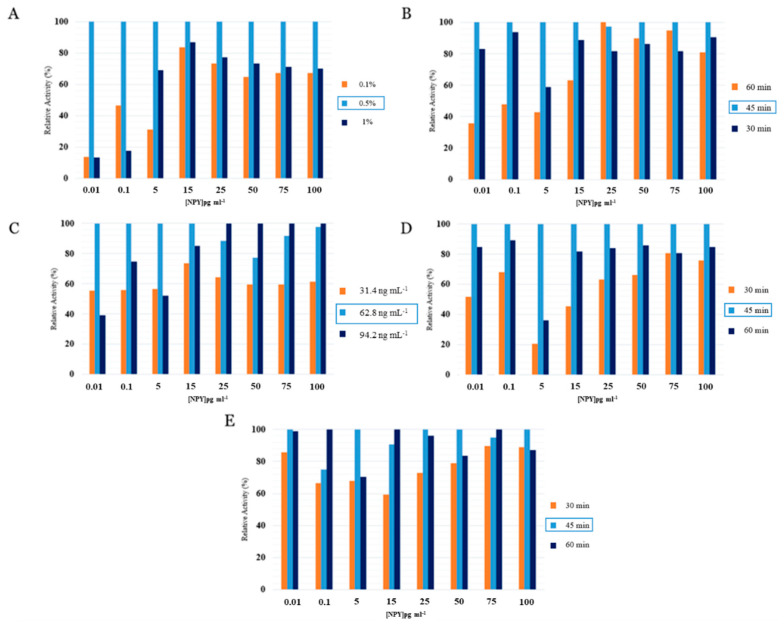
The optimization process and the optimal parameters chosen for the NPY immunosensor. (**A**) Optimum concentration of HMDC (0.5%); (**B**) Optimum incubation period for HMDC (45 min); (**C**) Optimum concentration of anti-NPY (62.8 ng mL^−1^); (**D**) Optimum incubation period for anti-NPY (45 min); (**E**) Optimum incubation time for NPY (45 min).

During optimization experiments, the EIS signals obtained by the biosensor system were compared in terms of their percentage activity. HMDC concentrations of 0.1 percent, 0.5 percent, and 1 percent were investigated to determine the optimal HMDC concentration, which was the initial step in optimizing the biosensor’s development. Figure 3A demonstrates that the EIS signals obtained at 0.1 percent concentration are the lowest. This concentration is deemed insufficient for the effective development of an immobilization layer on the surface of an electrode. It was hypothesized that when HMDC concentrations increased, EIS signals would increase. At a maximum concentration of 1 percent, it was shown that the NPY binding capacity reduced due to surface accumulation.

The ideal concentration of HMDC was confirmed to be 0.5%. After determining the appropriate HMDC concentration, 30, 45, and 60 min of incubation were tried. After 30 and 60 min of incubation, the anti-NPY/NPY interaction was reduced, and the ideal incubation time was determined to be 45 min. Optimization of the anti-NPY concentration was done using 31.4, 64.8, and 94.2 ng mL^−1^ concentrations. As seen in Figure 3C, the percentage of activity increased as antibody concentration increased, and comparable results were observed at high NPY concentrations of 64.8 and 94.2 ng mL^−1^. To construct a more efficient system, the ideal concentration value of 64.8 ng mL^−1^ was chosen. Then, 30 min, 45 min, and 60 min of antibody incubation were determined. The optimal value corresponded to the 45-min period with the highest anti-NPY/NPY interactions. Finally, the NPY antigen incubation duration was optimized. We assessed 30-, 45-, and 60-min incubation times. Figure 3E suggests that 30 min is insufficient for antibody–antigen interaction. However, it was discovered that the antibody’s capacity to bind NPY was reduced after 60 min of incubation compared to 45 min of incubation. It was established that the ideal incubation period was 45 min.

Figure 3 Illustrates the optimization process and the optimal parameters chosen for the NPY immunosensor, namely, (A) Optimum concentration of HMDC (0.5%); (B) Optimum incubation period for HMDC (45 min); (C) Optimum concentration of anti-NPY (62.8 ng mL^−1^); (D) Optimum incubation period for anti-NPY (45 min); (E) Optimum incubation time for NPY (45 min).

**Figure 4:**
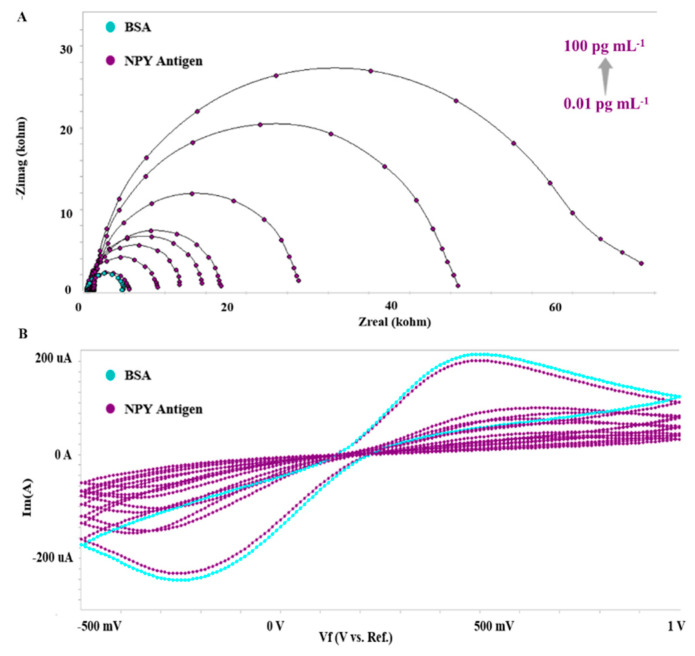
NPY concentration determination at increasing concentrations. (**A**) EIS spectra; (**B**) CV voltammograms.

Figure 4A,B show the EIS spectrum and CV voltammograms for the measurement of NPY at various concentrations between 0.01 and 100 pg mL^−1^ after the immobilization processes and optimization operations have been conducted. The calibration plot of the NPY biosensor was derived using impedimetric measurements computed by the following equation: ΔR_ct_ = R_ct_ (NPY antigen)- Rct (BSA). Rct (NPY antigen) indicates the diameter of the semicircle after the interaction between anti-anti-NPY and NPY antigen. While increasing antigen concentrations caused an increase in Rct, the peak currents of CV reduced as predicted.

**Figure 5:**
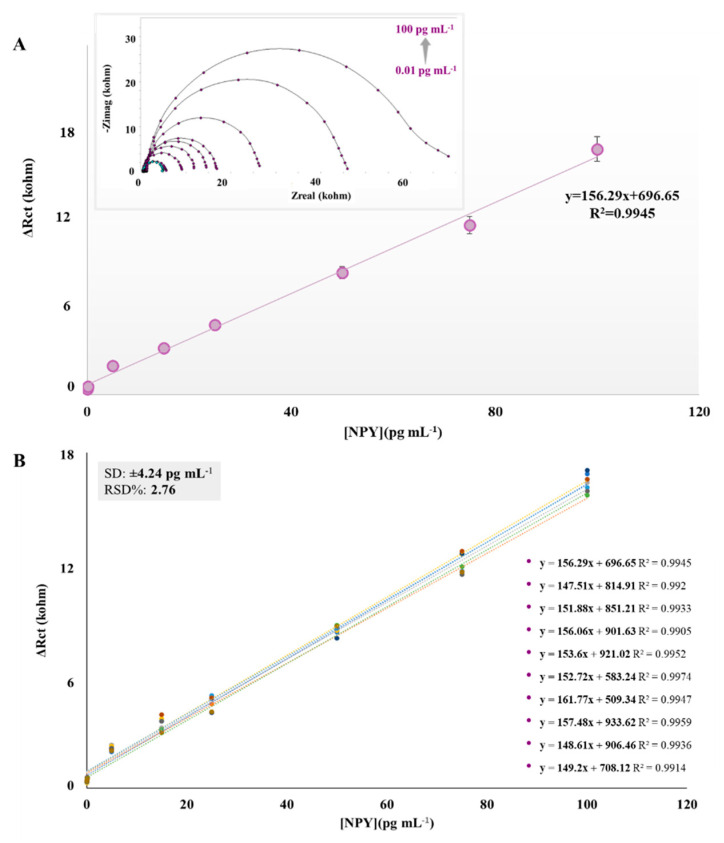
NPY immunosensor characterization investigations. (**A**) Calibration plot; (**B**) Reproducibility (n = 10).

The calibration graph for the proposed NPY biosensor is shown in Figure 5A. As shown, the suggested biosensor has a wide linear detection range of 0.01–100 pg mL^−1^. In addition, the LOD and LOQ values were calculated to be 0.0296 pg mL^−1^ and 0.0989 pg mL^−1^, respectively. The LOD and LOQ values are determined using the equation “k.S(standard deviation)/m (curve slope)” [13]. The k number for LOD was three and for LOQ it was ten. The repeatability of a biosensor is an important measure of its stability. Using the calibration equation, the mean value, standard deviation, and coefficient of variation were estimated to be 24 pg mL^−1^, 1.55 pg mL^−1^, and 2.39 percent, respectively. Consequently, it can be concluded that the created biosensor is extremely repeatable. 

**Figure 6:**
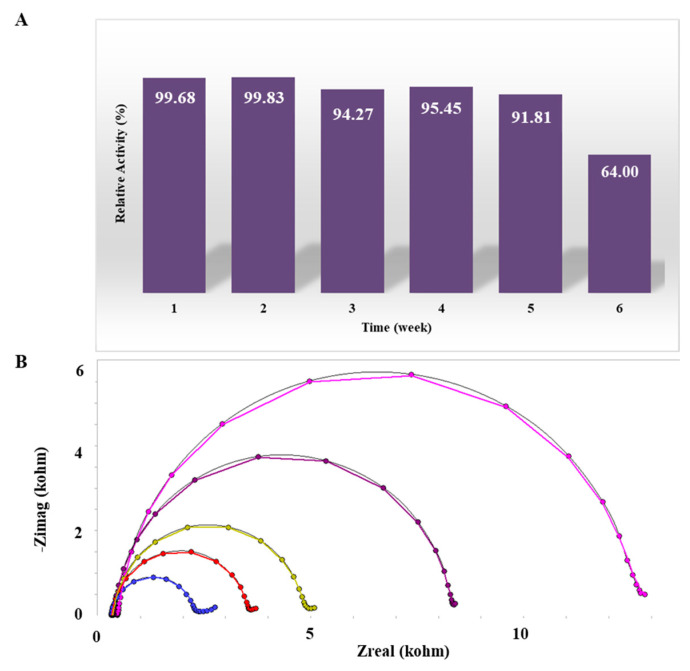
(**A**) Shelf Life of the NPY immunosensor; (**B**) Kramers–Kronig Transform characterization: blue (-●-): OH, red(-●-): OH/HMDC, yellow(-●-): OH/HMDC/Anti-NPY, purple(-●-): OH/HMDC/Anti-NPY/BSA, pink(-●-): OH/HMDC/Anti-NPY/BSA/NPY.

Graphs exhibiting the reproducibility of the proposed NPY biosensor system are shown in Figure 5B. To confirm reproducibility, NPY biosensors were manufactured by different persons under the same conditions and at different times, and impedance measurements were performed within the linear sensing range (0.01–100 pg mL^−1^). This process was repeated 10 times. The relative standard deviation of the overlapping line plots was calculated to be 3.07 percent. The reproducibility research conducted independently validates the designed NPY biosensor’s stability. The shelf life of NPY immunosensors has been evaluated. The ideal biosensor should have a lengthy shelf life. Therefore, measurements of the storage were conducted for five weeks. Electrodes made under the same conditions and at the same time were kept at +4 °C in a dark environment. Every week, one of the produced electrodes was incubated with 25 pg mL^−1^ NPY antigen for EIS measurement. During the storage period, it was noted that there was no major decline of NPY test activity (Figure 6A). With this novel immobilization method, it is possible to generate a stable biosensor surface. When Figure 6B is compared to the experimental data in the Kramers–Kronig transform, it is obvious that the virtual computation of the components matches with the experimental data. The transformation is used to analyze whether the constructed biosensor system’s impedance spectrum is modified by external factors and deviations [14].

Following analytical experiments, the suggested biosensor’s response to human serum samples was examined. The statistics in Table 1 include relative standard deviation and recovery. The results suggest that the error margin is minimal and that the proposed biosensor has high potential for detecting NPY in clinical situations.

**Figure 7:**
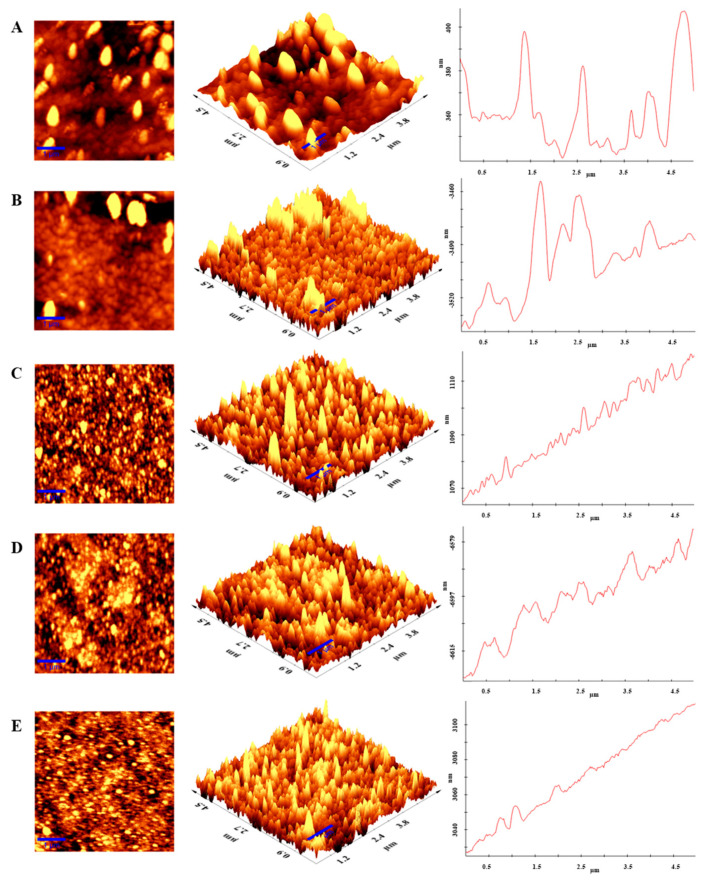
AFM images of NPY immunosensor. (**A**) Hydroxylation; (**B**) HMDC Treatment; (**C**) Anti-NPY; (**D**) BSA; (**E**) NPY antigen immobilization steps.

AFM was used to observe the surface morphologies of the changed electrodes. The morphological information produced by the AFM analysis provides a physical depiction of the electrode surface modification processes. The roughness value (Ra) seen in these images can be correlated with the biosensor’s surface properties. In these figures, surface roughness is shown by the distance between peaks and valleys. As seen in Figure 7A, the initial layer generated with hydroxyl groups for immobilization is reasonably uniform and has small peaks and valleys distance. On a scale of 5 × 5 µm, the average surface Ra of this platform is 37.891 nm. The surface displays positive skewness and high peaks after HMDC treatment (Figure 7B). Active end sites are appropriate surfaces for antibody immobilization. The surface structure of the working electrode changed following the covalent attachment of anti-NPY. As a result of the presence of antibodies, the peaks appear to assume more spherical shapes (Figure 7C) [15]. Due to the formation of the protein layer, Ra measures 356.598 nm. This clearly demonstrates that the surface roughness of the biosensor increases as surface modification proceeds. Ra was measured to be 166.689 nm based on AFM images captured following immobilization of the blocking agent BSA (Figure 7D). The decrease in the peak-to-valley distance at this stage may be the result of the blocking agent filling the active surfaces that are not involved in the binding. After applying NPY to the ITO-PET surface, the morphology of the electrode surface was studied in the last step. The AFM image reveals that the interaction of NPY and anti-NPY results in a substantial alteration in surface morphology (Figure 7E). The immobilization of NPY at the surface led to an increase in the surface’s density. After antigen–antibody interaction, the value of Ra (237.766 nm) increased. The surface morphology data support the conclusion that the biosensor was effectively produced since they are compatible with EIS and CV data.

## 4. Discussion

Using physical and biological techniques to identify biomarkers, new technologies that are rapid, precise, and sensitive are being studied. Due to the great sensitivity of biosensors as well as the simple, rapid, and inexpensive detection of analytes, this field has been one of the most intensely investigated in recent years. However, advancements in multiplexed detection, robustness, stability, and specificity are still required for precise and differentiated illness identification. As summarized in Table 2, a limited number of biosensor systems have been designed for NPY detection in studies conducted to date. The detection of NPY can be accomplished using a wide variety of biosensor approaches. Using guided-mode resonance (GMR) biosensor technology, Abdallah et al. demonstrated the quantitative detection of NPY biomarkers. The label-free sensor operates in the near-infrared area of the electromagnetic spectrum, which exhibits different resonance characteristics. The interaction of NPY with certain molecules on the sensor surface results in spectrum changes that directly and without extra processing identify the binding event. In this study, the sensor reached a detection limit of 0.1 pM NPY in the 0.1–10 pM detection range by employing the sandwich assay technique [16]. Churcher et al. developed a novel electrochemical detection technology for measuring NPY biomarker concentrations. The study compares non-porous and porous sensor platforms. This research employed a unique approach to creating an optimum nanobioelectronic interface for measuring NPY. The sensor’s detection limit is 10 pg mL^−1^, and its range is 20–500 pg mL^−1^ [17]. In a separate work, López et al. reported an aptamer-modified microelectrode for detecting Neuropeptide Y using EIS. This approach may detect NPY by adsorption at the surface of the microelectrodes, with the specificity given by aptamer functionalization of the microelectrode surface. A linear range from 10 to 1000 ng mL^−1^ of NPY was obtained [18]. Das et al. designed a silicon nitride (SiN) microring-resonator sensor for detecting neuropeptide Y protein. The lowest detectable concentration was calculated as 1 µg mL^−1^ (~235 nM). Experimentally, they demonstrated the expansion of detection to nanomolar concentration levels in a standard assay system with a sensitivity of 0.1 nm (g mL^−1^) ^−1^ [19]. For label-free NPY detection, Fernandez et al. proposed an electrokinetically improved aptamer sensing platform on a disposable plastic chip. The sensor comprises of aptamer-functionalized graphene-gold nanocomposites (Gr-AuNs) etched within a nanoslit on cyclic olefin copolymer using nanoimprint lithography. NPY can be identified at 10 pM LOD, and its concentration range is 10–1000 pM [20].

In this biosensor, which we developed based on antibody–antigen-specific interaction, NPY protein can be detected successfully and practically without a crosslinker or marker. The developed NPY biosensor stands out with its extremely low LOD (0.02968 pg mL^−1^) and a very wide detection range (0.01–100 pg mL^−1^). The acquired degree of sensitivity places the suggested biosensor among the most sensitive biosensing devices in the scientific literature. In addition to enhanced precision, the suggested biosensor offers good reproducibility, repeatability, and storage stability. It is a highly practical method with a two-day preparation process and a marker measurement that takes only minutes to complete. In light of these characteristics, it may be concluded that the proposed biosensor offers tremendous promise for clinical applications involving NPY analysis.

## 5. Conclusions

In this report, an electrochemical immunosensor with a broad detection range and high sensitivity was constructed to detect NPY by activating the ITO-PET surface with HMDC. The cyanate terminals of HMDC can react rapidly with OH polyol groups and functional groups present in all proteins. The proposed NPY biosensor has a wide linear detection range (0.01–100 pg mL^−1^), a low LOD (0.02968 pg mL^−1^), and a low LOQ (0.0989 pg mL^−1^). The measurement of NPY using this unique detection approach is extremely robust and reproducible. In future research, these established immobilization methods will make it possible to create an electrochemical immunosensor with a wider detection range for a variety of biomarkers. 

## Figures and Tables

**Table 1:** The NPY biosensor’s applicability to human serum samples.

Serum Sample Number	NPY Conc. (pg mL^−1^)	Standard Added Conc. (pg mL^−1^)	Measured Conc. (pg mL^−1^, n = 3)	RSD (%) (n = 3)	Recovery (%)
1	66.22	5	69.09/72.16/71.47	0.44	99.56
		25	90.62/95.09/93.39	1.95	101.95
2	21.84	5	26.13/26.31/28.51	0.53	100.53
		25	48.29/46.70/47.40	1.31	101.31
3	0.82	5	5.59/7.21/4.5	0.92	99.08
		25	25.69/24.22/23.90	4.94	95.06
4	5.44	5	9.08/10.00/8.83	12.18	87.82
		25	27.77/28.12/29.52	6.90	93.1
5	18.17	5	22.59/23.41/24.58	1.51	101.51
		25	43.34/46.18/42.70	2.05	102.05

**Table 2:** Summary of various biosensor systems designed for NPY detection.

Transducer Type	The Detection Range	Limit of Detection	Reference
Guided-mode resonance (GMR)	0.1–10 pM	0.1 pM (0.4 pg mL^−1^)	[16]
Electrochemical	20–500 pg mL^−1^	10 pg mL^−1^	[17]
Electrochemical	10 to 1000 ng mL^−1^	-	[18]
Microring resonator	-	1 µg mL^−1^	[19]
Electrochemical	10–1000 pM	10 pM (~40 pg mL^−1^)	[20]
Electrochemical	0.01–100 pg mL^−1^	0.02968 pg mL^−1^	This study
